# Comparing the effect of in-person and virtual childbirth preparation trainings on the fear of childbirth (FOC) and pregnancy experience of pregnant women: protocol for a quasi-experimental feasibility study

**DOI:** 10.1186/s40814-021-00933-w

**Published:** 2021-11-05

**Authors:** Seyedeh Robab Mousavi, Leila Amiri-Farahani, Syedeh Batool Hasanpoor-Azghady, Soghra Omrani Saravi

**Affiliations:** 1 Department of Reproductive Health and Midwifery, School of Nursing and Midwifery, Iran University of Medical Sciences, Tehran, Iran; 2 Department of Reproductive Health and Midwifery, Nursing Care Research Center (NCRC), School of Nursing and Midwifery, Iran University of Medical Sciences, Tehran, Iran

**Keywords:** Childbirth preparation, Fear of childbirth, Pregnancy experience, Type of delivery, Virtual education, Feasibility study

## Abstract

**Background:**

Childbirth preparation trainings are an important component of prenatal education, and pregnant women are increasingly interested in seeking information from online sources. The aim of this study is to compare the feasibility and the effects of in-person and virtual childbirth preparation training courses on the pregnancy experience, fear of childbirth (FOC), birth preference, and type of delivery among pregnant women.

**Methods:**

In total, 165 primiparous women referring to a prenatal clinic at Milad Hospital in Tehran, will be included in this study. The subjects will be selected using the convenience sampling method and will be divided into three groups of study A, study B, and control. The study groups A and B will receive virtual and in-person childbirth training with similar content, respectively. The control group will receive only routine prenatal care. In the 18th and 20th weeks of pregnancy, the demographic information, pregnancy experience scale (PES), and version A of the Wijma delivery expectancy/experience questionnaire (WDEQ-A) will be completed, and in the 36th and 38th weeks of pregnancy, the PES and WDEQ-A questionnaires, as well as birth preference form will be completed. The type of delivery will be recorded in the first few days of postpartum.

**Conclusion:**

This quasi-experimental clinical trial will investigate the effect of virtual childbirth preparation training on primiparous women. The expected outcomes will include the difference in pregnancy experience measured by the brief version of PES, the difference in FOC measured by WDEQ-A, the birth preference, and the type of delivery.

**Trial registration:**

IRCT.ir: IRCT20180427039436N2;

**Supplementary Information:**

The online version contains supplementary material available at 10.1186/s40814-021-00933-w.

## Introduction

One of the most important goals of the World Health Organization is to achieve integrated and high-quality pregnancy care services to create positive pregnancy and childbirth experiences in women [1]. Childbirth preparation training courses prepare pregnant women for childbirth, and they are conducted individually or in a group. The ultimate goal of these courses is to improve women’s lifestyle during pregnancy and labor, and also after the childbirth while preserving the neonatal and maternal rights [2]. The advantages of prenatal pregnancy include eliminating pregnancy and delivery misconceptions, improving mother’s self-confidence regarding labor and childbirth, empowering women to select a safe delivery method, and reducing the need for analgesics during labor and childbirth [3]. On the other hand, inadequate or lack of prenatal care leads to the tendency toward cesarean section, postpartum depression, and challenges in accepting the maternal role [4].

Both pregnancy and childbirth lead to various mental and physical changes that can cause pregnant women to simultaneously experience pleasant and unpleasant feelings. On the one hand, changes, such as feeling fetal movements, thinking about the fetus, and speaking with the spouse about the newborn’s name are among the pleasant experiences during pregnancy. On the other hand, seeing common pregnancy complications in media or reading about it, and thinking about delivery and health of the neonate, especially as the due date gets closer, increases the anxiety and stress of pregnant women. Learning about changes and ways to overcome them through childbirth preparation trainings can affect the pregnant women’s pregnancy and delivery experiences and the selection of a safe delivery method [5, 6].

Studies on the effectiveness of childbirth preparation trainings have produced conflicting results [7–9]. One study showed that these trainings significantly reduce the fear of childbirth (FOC) and increase self-efficacy in pregnant women [7]. In contrast, another study found no significant effect of these trainings on reducing anxiety and increasing maternal self-efficacy [8]. Moreover, it has been reported that these trainings play a role in reducing childbirth anxiety, but have no effects on reducing the duration of labor and type of delivery [9].

Despite the efforts made to decrease the prevalence of cesarean section, this delivery method accounts for 48% of all deliveries in Iran, which is extremely above the global standard rate [10]. Given the fact that one of the important goals of childbirth preparation training courses is to reduce the rate of cesarean section, achieving this goal might and also proving the effectiveness of these trainings may require further investigations. According to previous studies, the quality of educational content and its presentation method play a significant role in the effectiveness of childbirth preparation trainings [9].

Various healthcare education methods, such as face-to-face teaching and using pamphlet, video clip, and mass media for education have been used for decades to motivate healthy behaviors in pregnant women. Nevertheless, women have increasingly become interested in using digital sources and internet to obtain information on pregnancy [11, 12]. Moreover, internet-based learning has made it possible for learners to exchange personal experiences and establish interpersonal relationship. Also, among virtual education methods, social media-based learning has been increasingly used worldwide. In Iran, the Telegram application ranks 14 among the most popular social media [11].

One of the important educational principles in healthcare is the selection of an appropriate education method to maximize the active participation of individuals [13]. Education through social media plays an important role in teaching and forming positive behaviors at the community level [14]. Some of the advantages of social media include the provision of services, such as health-related information at any time and place, online consultation, health-related question and answer, interactions between users, emotional and social support, and easy access regardless of the age, level of education, race, and place of residence [15]. Furthermore, social media are effective in shaping the attitudes of individuals by creating an interactive environment among group members [11].

Despite the rapid development of social media and their ever increasing popularity among members of the society, especially pregnant women, little research has been conducted on the virtualization of childbirth preparation training courses. In a study of teaching exclusive breastfeeding methods through Telegram social media, the mothers stated that Telegram-based education was more useful than in-person participation in the breastfeeding training courses, they also believed that Telegram social media is an effective educational tool in the field of healthcare [11]. It has also been shown that e-learning can increase the level of satisfaction and awareness of primiparous women regarding prenatal care, compared to educational booklets [16]. Although these results are promising, it is not clear whether virtualize interventions will be feasible for childbirth preparation training in Iran.

An investigation of childbirth preparation training courses showed that these courses have had little effect on pregnancy outcomes in the past few years, since the rate of cesarean sections is still high in Iran [3]. This can be attributed to factors, such as the lack of awareness and unwillingness of pregnant women to participate in natural childbirth training courses, overcrowded classes at hospitals, lack of time (especially among employed women), living far away from centers that offer such trainings, and heavy urban traffic jam, especially in densely populated cities such as Tehran.

Although attending childbirth preparation classes seems beneficial for all pregnant women, these classes are not very popular in Iran. Also, none of the studies available on e-learning in the literature has specifically targeted pregnant women, who probably would benefit the most from the lifestyle changes. Due to the importance of childbirth preparation trainings among pregnant women and ease of access to internet and virtual communication devices such as smartphone in Iran, the present study will be conducted to investigate the feasibility and the effect of childbirth preparation training courses using two in-person and virtual education methods on the pregnancy experience, FOC, birth preference, and type of delivery in pregnant women.

The primary objective of study is the feasibility of recruitment and feasibility of intervention adherence of participants in both in-person and virtual education groups. The secondary objectives are the effect of childbirth preparation training courses using two in-person and virtual education methods on the pregnancy experience, FOC, birth preference, and type of delivery in pregnant women.

## Methods

### Setting

The required data will be collected from childbirth preparation training courses that are regularly held in Milad Hospital, Tehran, Iran. These courses have high educational quality and are based on a precise schedule, according to the educational plans defined by the Ministry of Health and Medical Education of Iran. Notably, these courses are held in classes that contain 10-12 individuals at the prenatal clinic of Milad Hospital, which is one of the main referral hospitals in Tehran province.

At Milad Hospital, where all participants in the three groups will be selected from, it is mandatory for pregnant women to attend childbirth preparation classes in order to deliver their children at this hospital, so women who wish to give birth in this hospital have to participate regularly in these classes (participating in six sessions from eight at least). The samples in the virtual and in-person intervention groups will be selected among these women. There is another group of women who only visit a specialist or midwife to receive a pregnancy care and advice, and do not want to give birth at Milad Hospital or attend the preparation classes. These people will be used for the control group.

### Inclusion and exclusion criteria

All eligible pregnant primiparous women between the ages of 18 and 35 years who refer to the hospital for prenatal care at 18-20 weeks of gestation will be requested to participate in the study. Inclusion criteria are having Iranian nationality, having low-risk pregnancy^1^, having no history of infertility and mental diseases, having the ability to read and write, and having smartphone or computer with access to internet and Telegram messaging application.

After the sampling, participants who go into preterm labor, show any signs of high-risk pregnancy, wish to participate in other in-person or virtual childbirth preparation classes than the ones offered at Milad Hospital, and are unwilling to continue with the study will be excluded from the study. However, the educational content will be provided to the excluded samples that are still eager to receive them. In addition, the participants in the study group A who do not send any feedback through Telegram, and the participants in the study group B who do not attend more than two training sessions will be excluded from the study.

### Research design and sampling

**Fig. 1:**
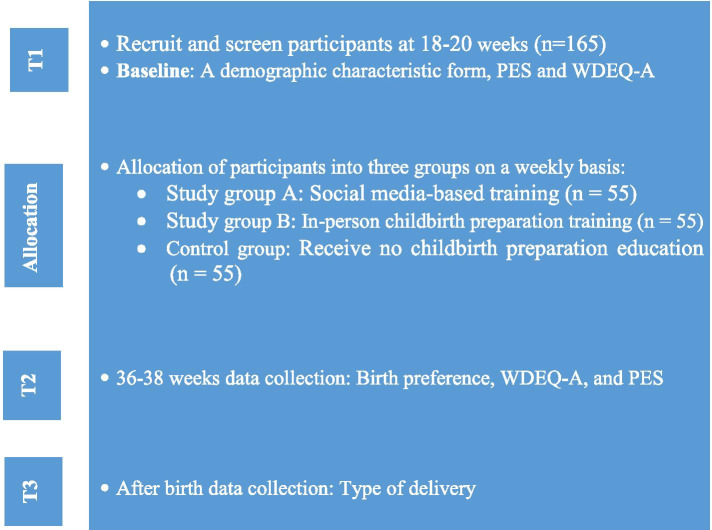
Key points for data collection

This feasibility quasi-experimental study with a control group was reported in accordance with the Consolidation Standards of Reporting Trials (CONSORT) statement for pilot and feasibility trials (Additional file 1) [17]. The current study will be conducted on 165 primiparous women with 18-20 gestational weeks that will be selected by convenience sampling method. The researcher will attend the prenatal clinic for sampling on a daily basis. The participants will be equally allocated into two studies and one control groups (Fig. 1). The participants in study groups A and B will receive virtual and in-person childbirth preparation trainings, respectively. Also, the control group will receive no education on childbirth preparation during pregnancy.

### Intervention

In the study group A, a Telegram room entitled: “Virtual childbirth preparation training courses” will be created for uploading the educational content. In addition, another Telegram room will be created with the same name in which, the participants could ask their questions and receive answers. The content of virtual education will be provided based on the standard regulations of Iran and also, the Mayer’s principles of multimedia learning [18] using text, picture, video clips, podcast, and MPEG4 (MP4) videos. Moreover, the maximum size of educational files will be 50 MB in the form of 5-15 min long videos.

To increase the quality of training and prevent sending the whole content at once, the content of each session will be provided in relevant files that will be uploaded at a specific time (except for Thursdays and Fridays). In total, the educational contents will include six PDF files; 21 videos; four podcasts about relaxation and breathing techniques during labor; 14 video clips; and 64 MP4 video files about exercises during and after pregnancy, massage, labor positions, birth ball exercises, and postpartum exercises. The contents will be uploaded directly on the Telegram rooms as shown in Table 1.

**Table 1:** The educational content of virtual and in-person childbirth preparation training courses for the study groups A and B

Time	Content	Objectives	Uploading the virtual content to the Telegram room
First session: 20-23 gestational weeks	-Personal hygiene with an emphasis on anatomy and physiology during pregnancy	-An introduction to the reproductive system -Changes and adaptations of the body during pregnancy, common complaints and coping strategies -Personal hygiene	4 video clips for theoretical content 9 videos (1) for exercises during pregnancy 1 podcast for relaxation (1)
Second session: 24-27 gestational weeks	-Pregnancy diet	-Pregnancy diet with emphasis on what to eat -An introduction to the food pyramid	1 video clip for theoretical content 9 videos (2) for exercises during pregnancy Repeating relaxation (1)
Third session: 28-29 gestational weeks	-Mental health during pregnancy	-An introduction to the fetal growth and development -Preparing for motherhood -Preparing for fatherhood	2 video clips and 1 PDF file for theoretical content 9 videos (3) for exercises during pregnancy Repeating relaxation (1)
Fourth session: 30-31 gestational weeks	-Risk factors during pregnancy	-Learning the signs of preterm childbirth and how to react to them	1 video clip for theoretical content 9 videos (4) for exercises during pregnancy 1 podcast for relaxation (2)
Fifth session: 32-33 gestational weeks	-Planning for delivery and selecting the type of delivery	-Natural delivery vs. cesarean section -Different pain control methods during labor -Selecting the location and necessary equipment for delivery	2 video clips and 1 PDF file for theoretical content Repeating relaxation (2) Visiting the delivery room in person
Sixth session: 34-35 gestational weeks	-An introduction to vaginal delivery	-An introduction to birth hormones -An introduction to labor stages and self-care at each stage	1 video clip, 1 PDF file, and 2 videos for theoretical content 11 videos for labor and childbirth 1 podcast for breathing techniques 1 podcast for relaxation techniques (3)
Seventh session: 36 gestational weeks	-Postpartum care and breastfeeding	-Postpartum care and recognition of dangerous symptoms -An introduction to breastfeeding methods and breast diseases -An introduction to postpartum exercises	1 video clip, 1 PDF file, and 8 pictures for theoretical content 8 videos for massage during pregnancy and labor Repeating relaxation (3)
Eighth session: 37 gestational weeks	-Neonatal care	-Neonatal care and risk factors	2 PDF files and 2 videos for theoretical content 9 videos for postpartum exercises Repeating relaxation (1)

The questions asked on the Telegram room will be answered by the researcher at least twice a day. During the last weeks of pregnancy and after uploading all files, the educational content regarding labor and childbirth preparation methods will be reviewed. To ensure the accurate learning of breathing and relaxation techniques, two 2-h long sessions will be held for the in-person training of the study group A at the hospital along with the fifth and eighth sessions of the study group B. Also, to ensure that the messages are read by the participants, before joining the group, they will be asked to share their last seen status with the researcher. Moreover, they will be required to be online at least once a day to watch the videos or read the messages sent. The last seen time of the participants will be controlled by the researcher, and those who are not online, have not seen the messages, or have not given feedback for 7 days will be contacted by message at first. If no response is received from them, the researcher will contact them through a direct phone call to ask their reasons for not responding the messages. If needed, another cell phone number will be obtained to re-send the Telegram messages to that number. Otherwise, the subject will be excluded from the study.

On the other hand, subjects in the study group B will participate in eight 2-h long sessions during the 20-37 weeks of gestation. The educational sessions will be held at the hospital based on the guidelines of the Ministry of Health. In each session, 1 h will be allocated to teaching theoretical content followed by 45 min of teaching stretching, breathing, and relaxing techniques, as well as practical strategies for posture correction and massage therapy education. The participants will be given 15 min to ask any questions, which will be answered by the researcher.

The teaching instructors for the training sessions will be midwives who are certified as childbirth educators. If needed, they will use textbooks, video and audio files, music, posters, medical moulages, whiteboard, and slides. Table 1 presents the content, time, and objectives of the educational sessions that are similar for both study groups. To increase the generalizability of the results, another group will be considered as the control group that receives no education about pregnancy and childbirth preparation. It is important to note that all three groups will receive the routine prenatal care provided at the hospital. All participants will also be followed up until delivery.

### Measurements and feasibility outcomes

The primary outcome of the present study would be the study feasibility that will be assessed based on recruitment and intervention adherence for all of the participants in both groups.

The secondary outcome of the present study would be the improvement of pregnancy experience measured by the brief version of the pregnancy experience scale (PES) [19]. The secondary outcome would be the decreased FOC measured by the version A of Wijma Delivery expectancy/experience questionnaire (WDEQ-A) [20]. In addition, the other outcomes would be the birth preference and type of delivery, respectively. Data collection is shown in the flow diagram (Fig. 1). Data will be collected at 3 times by self-reporting method through Telegram messages: Recruitment (demographic characteristics questionnaire, PES, WDEQ-A) ≈ 18-20 weeks (T1); 36-38 weeks (PES, WDEQ-A, and birth preference) (T2); the first few days of postpartum (type of delivery) (T3).

### Sample size

A useful introduction to feasibility and pilot studies is given by Sim and Lewis, who recommend at least 50 participants. Therefore, according to strong evidence, sample size 50 is considered in current study [21–23].

To determine the sample size for other variables in the present study, the sample size has been calculated with a test power of 80% and a significance level of 0.05. Moreover, it is considered that in-person and social media-based education could affect the type of delivery among the study groups, leading to a 25% decrease in cesarean sections, compared to the control group. By using the effect size equation and considering 10% sample drop, the total sample size has been estimated at 55 participants per group. It should be noted that the ratio of primiparous women undergoing a cesarean section in Iran is assumed to be 0.55 [24] based on the statistics, so the sample size is large enough to measure all variables in this study.

$$n=\frac{{\left({z}_{1-\alpha\left/ 2\right.}\sqrt{2\overline{p}}\overline{q}+{z}_{1-\beta}\sqrt{p_1{q}_1+{p}_2{q}_2}\right)}^2}{{\left({p}_1-{p}_2\right)}^2}=\frac{{\left[\left(1.96\times \sqrt{2\times 0.42\times 0.57}\right)+\left(0.84\times \sqrt{\left(0.55\times 0.45\right)+\left(0.3\times 0.7\right)}\right)\right]}^2}{{\left(0.55-0.3\right)}^2}=50$$

$$\overline{p}=\frac{p_1+{p}_2}{2}$$

The sample size for the other variable of the study (fear of childbirth) was determined to be 43 participants with the confidence level of 95%, the power test of 80%, and based on the Toohil et al. study standard deviation for intervention and control group was 13.1 and 9.7, respectively with *d* = 7 [25], which was less than the sample size calculated based on the type of delivery. The sample size for the other two variables (childbirth experience and birth preference) was less than the one calculated for the above variables.

$$n=\frac{{\left({z}_{1-\alpha\left/2\right.}+{z}_{1-\beta}\right)}^2\times \left({s}_1^2+{s}_2^2\right)}{d^2}$$

$$n=\frac{{\left(1.96+0.84\right)}^2\times \left({9.7}^2+{13.1}^2\right)}{7^2}\approx 43$$

### Data collection

The demographic characteristics questionnaire will be developed in two sections. The first part is related to personal information, such as age, level of education, and occupational and economic status of couples. The second part is related to the history of pregnancy, such as the date of the last menstruation period, the expected date of delivery, and the recent pregnancy status.

The brief version of PES was first developed by DiPietro (2008) with 20 items. The first and second 10 items assess the uplifts and hassles among pregnant women. The items are scored based on a 4-point Likert scale ranging from not at all (0) to somewhat (1), quite a bit (2), and a great deal (3). The total scores for each section are within the range of 0-30, and the higher scores indicate higher levels of uplifts and hassles. The reliability of the tool has been measured with internal consistency of 0.82 and 0.83 for uplifts and hassles, respectively using Cronbach’s alpha [19]. In Iran, the Persian translation of this tool was carried out by Hajifoghaha, while its psychometric properties were determined by Ebadi. The face, content, and construct validity of PES have also been confirmed. In order to determine the reliability of the test, an internal consistency with a Cronbach alpha of 0.777 and 0.672 was used for uplifts and hassles, respectively. The intra-class correlation coefficients of 0.712 and 0.672 have also been used for uplifts and hassles, respectively. This tool is valid during 15-38 gestational weeks [26].

The WDEQ-A was developed by Wijma (1998) and consists of 33 items which are scored based on a 6-point Likert scale ranging from “not at all…” to “extremely…” The total scores obtained are within the range of 0-165, so that the scores of ≤ 37, 38-65, 66-84, and ≥ 85 are recognized as mild, moderate, severe, and clinical fear, respectively [27]. Notably, reverse scoring is applied to items 2, 3, 6, 7, 8, 11, 12, 15, 19, 20, 24, 25, 27, and 31. The reliability of the results during the last trimester has been estimated at 0.89 by Cronbach’s alpha, and the split-half reliability of the test has been obtained at 0.91 [20]. The Persian version of this questionnaire was developed by Abedi et al. in Iran, and the reliability of this scale has been reported at 0.64 for the whole pregnancy period using Cronbach’s alpha [26].

### Analytic plan

Feasibility of recruitment will be achieved if ≥ 30% of eligible participants enroll in the study, and ≥ 80% participants complete follow-up assessments at 36-38 weeks (PES, WDEQ-A, and birth preference) (T2); the first few days of postpartum (type of delivery) (T3). Feasibility of intervention adherence will be achieved if average proportion of intervention adherence rate is ≥ 0.8 (participating in six sessions from eight at least for both the virtual and in-person childbirth preparation training groups).

The collected data will be analyzed in the SPSS software (version 19) using descriptive statistics, such as frequency distribution tables. Also, the Chi-square test and one-way analysis of variance (ANOVA) will be used to compare the quantitative and qualitative variables in three groups, respectively. In addition, the paired *t* test will be employed for intragroup comparison. The *p* value of less than 0.05 will be considered statistically significant.

### Ethical considerations

The study protocol has been approved by the Ethics Committee of Iran University of Medical Sciences, Tehran, Iran (IR.IUMS.REC1396.9511373011), and has also been registered in the clinical trial center of Iran (IRCT20180427039436N2). All participants will be informed about the research objectives and process, as well as the confidentiality of their personal information and then a written informed consent will be obtained from them. In addition, pregnant women will be informed that they have the right to withdraw from the study at any time and still receive normal childbirth care. The research procedure will impose no costs on participants, and they will be provided with free services. In the present study, one code will be assigned to each sample, so the participants’ information will be entered into informational software anonymously. It should be noted that the researcher and all instructors of childbirth preparation trainings have participated in a 60-h long instruction training course held by the Ministry of Health and have obtained childbirth educator certification.

## Discussion

This quasi-experimental clinical trial with a control group aims to evaluate the feasibility and the effect of virtual and in-person childbirth preparation trainings on primiparous women. This study will be conducted by the reproductive health department of the Faculty of Nursing and Midwifery of Iran University of Medical Sciences, at the prenatal clinic of Milad Hospital in Tehran. The main results of the current study would include the improved experience of pregnancy, decreased FOC, increased rate of vaginal delivery, and reduced costs of healthcare for pregnant women.

It has been a decade since the first childbirth preparation training courses were held in Iran [28]. Nevertheless, the rate of cesarean section is still higher than the global standard [10]. The most important reason for the cesarean section is the fear of vaginal delivery due to a lack of knowledge and information about pregnancy and delivery [3]. Meanwhile, the results of a systematic review and meta-analysis revealed that educational interventions and self-hypnosis can significantly reduce the fear of childbirth. These results also suggest that educational interventions may reduce the fear of childbirth for twofold. The findings highlights the role of antenatal education in enhancing childbirth expectations and experiences [29].

Lack of knowledge, crowded childbirth preparation classes held at hospitals, and heavy urban traffic jam, especially in populated cities of Iran, have negative effects on the tendency of pregnant women to participate in natural childbirth preparation courses. In addition, other factors, such as the quality of educational content and the teaching methods used by different educators can play a significant role in the effectiveness of these courses [9]. With this background in mind, it is necessary to develop a suitable method according to the new educational approaches with the same content and easier access to provide pregnant women with health-related information they need.

This study evaluates an educational intervention that is based on the social media. The educational contents used in this study are based on standard in-person education confirmed by the Iranian Ministry of Health and Medical Education [28]. In addition, the intervention is provided online, which facilitates access to the educational contents at different times and locations, thereby increases the self-confidence of pregnant women and empowers them during critical stages of pregnancy [16]. The improvement of pregnancy experiences, especially reduced levels of FOC, and the development of a positive attitude toward labor are vital strategies for the promotion of vaginal delivery and reduction of cesarean section rate [30]. This feasibility trial will also provide insight for future studies concerning the promotion of health and well-being among women. Lessons learned from this study will be used to further refine the intervention and apply for a large-scale grant. By doing so, we can reduce the costs of healthcare and improve the quality of life of women during pregnancy, after labor, and during child development.

## Supplementary Information

**Additional file 1:** CONSORT checklist.

**Additional file 2:** TiDieR checklist.

## Data Availability

Not applicable

## Abbreviations

FOC: Fear of childbirth
PES: Pregnancy experience scale
WDEQ-A: Wijma delivery expectancy/experience questionnaire-version A
ANOVA: Analysis of variance

## Footnotes

1 This means that there are no active complications and there are no maternal or fetal factors that place the pregnancy at increased risk of complications. In contrast, a “high-risk” pregnancy means a woman has one or more issues that increase her—or her baby’s—chances for health problems or preterm delivery.

## References

[1] WHO. WHO recommendations on antenatal care for a positive pregnancy experience 2016 [Available from: http://apps.who.int/iris/bitstream/10665/250796/1/9789241549912eng.pdf?ua1. 28079998

[2] Krysa J, Iwanowicz-Palus GJ, Bień AM, Rzońca E, Zarajczyk M (2016). Antenatal classes as a form of preparation for parenthood: analysis of benefits of participating in prenatal education. Pol J Public Health.

[3] Masoumi SZ, Kazemi F, Oshvandi K, Jalali M, Esmaeili-Vardanjani A, Rafiei H (2016). Effect of training preparation for childbirth on fear of normal vaginal delivery and choosing the type of delivery among pregnant women in Hamadan, Iran: a randomized controlled trial. J Family Reprod Health.

[4] Gungor I, Beji NK (2012). Development and psychometric testing of the scales for measuring maternal satisfaction in normal and caesarean birth. Midwifery..

[5] Davoudi Z, Khodabakhshi-Kolaee A, Falsafinejad M (2018). The effectiveness of training of self-help program toward the parenthood on worry in pregnancy period among the nulliparous women. J Health Literacy.

[6] Hajifoghaha M, Ebadi A, Kariman N (2018). Persian translation of the pregnancy experience scale (PES)–brief version: confirmatory factor analysis. Methods. Pak J Med Health Sci.

[7] Serçekuş P, Başkale H (2016). Effects of antenatal education on fear of childbirth, maternal self-efficacy and parental attachment. Midwifery..

[8] Khaikin R, Marcus Y, Kelishek S, Balik C (2016). The effect of childbirth preparation courses on anxiety and self-efficacy in coping with childbirth. Clin Nurs Stud.

[9] Artieta-Pinedo I, Paz-Pascual C, Grandes G, Remiro-Fernandezdegamboa G, Odriozola-Hermosilla I, Bacigalupe A (2010). The benefits of antenatal education for the childbirth process in Spain. Nurs Res.

[10] Azami-Aghdash S, Ghojazadeh M, Dehdilani N, Mohammadi M (2014). Prevalence and causes of cesarean section in Iran: systematic review and meta-analysis. Iran J Public Health.

[11] Ghaffari M, Rakhshanderou S, Mehrabi Y, Tizvir A (2017). Using social network of TELEGRAM for education on continued breastfeeding and complementary feeding of children among mothers: a successful experience from Iran. Int J Pediatr.

[12] Daly LM, Horey D, Middleton PF, Boyle FM, Flenady V (2018). The effect of mobile app interventions on influencing healthy maternal behavior and improving perinatal health outcomes: systematic review. JMIR mHealth uHealth.

[13] Savabi Esfahani M, Kohan S, Ehsanpour S (2016). Promoting breastfeeding self-efficacy through role-playing in pregnant women. Int J Pediatr.

[14] Kohan S, Heidari Z, Keshvari M (2016). Iranian women’s experiences of breastfeeding support: a qualitative study. Int J Pediatr.

[15] Moorhead SA, Hazlett DE, Harrison L, Carroll JK, Irwin A, Hoving C. A new dimension of health care: systematic review of the uses, benefits, and limitations of social media for health communication. J Med Internet Res. 2013;15(4):e1933. PMID: 23615206. 10.2196/jmir.1933 PMC3636326 23615206

[16] Mohamadirizi S, Bahadoran P, Fahami F (2014). Effect of E-learning on primigravida women’s satisfaction and awareness concerning prenatal care. J Educ Health Promot.

[17] Eldridge SM, Chan CL, Campbell MJ, Bond CM, Hopewell S, Thabane L (2016). CONSORT 2010 statement: extension to randomised pilot and feasibility trials. BMJ..

[18] Clark RC, Mayer RE (2016). E-learning and the science of instruction: proven guidelines for consumers and designers of multimedia learning. Canada.

[19] DiPietro JA, Christensen AL, Costigan KA (2008). The pregnancy experience scale–brief version. J Psychosom Obstet Gynecol.

[20] Wijma K, Wijma B, Zar M (1998). Psychometric aspects of the W-DEQ; a new questionnaire for the measurement of fear of childbirth. J Psychosom Obstet Gynecol.

[21] Lancaster GA, Dodd S, Williamson PR (2004). Design and analysis of pilot studies: recommendations for good practice. J Eval Clin Pract.

[22] Sim J, Lewis M (2012). The size of a pilot study for a clinical trial should be calculated in relation to considerations of precision and efficiency. J Clin Epidemiol.

[23] Julious SA (2005). Sample size of 12 per group rule of thumb for a pilot study. Pharm Stat J Appl Stat Pharm Ind.

[24] Badiee S, Ravanshad Y, Azarfar A, Dastfan F, Babayi S, Mirzayi N (2013). Survey of cesarean deliveries and their causes in hospitals affiliated to Mashhad University of Medical Sciences, Iran, 2011. Iranian J Obstetr Gynecol Infertil.

[25] Toohill J, Fenwick J, Gamble J, Creedy DK, Buist A, Turkstra E (2014). A randomized controlled trial of a psycho-education intervention by midwives in reducing childbirth fear in pregnant women. Birth..

[26] Abedi P, Hazeghi N, Afshari P, Fakhri A (2016). The validity and reliability of Persian version of Wijma delivery expectancy/experience questionnaire (version a) among Iranian nulliparous women. Global J Health Sci.

[27] Zar M, Wijma K, Wijma B (2001). Pre-and postpartum fear of childbirth in nulliparous and parous women. Scand J Behav Ther.

[28] MOHME (2017). Prenatal and childbirth educations.

[29] Hosseini VM, Nazarzadeh M, Jahanfar S (2018). Interventions for reducing fear of childbirth: a systematic review and meta-analysis of clinical trials. Women Birth.

[30] Fenwick J, Gamble J, Creedy DK, Buist A, Turkstra E, Sneddon A (2013). Study protocol for reducing childbirth fear: a midwife-led psycho-education intervention. BMC Pregnancy Childbirth.

