# Supplementary material for: Comparing the effect of in-person and virtual childbirth preparation trainings on the fear of childbirth (FOC) and pregnancy experience of pregnant women: protocol for a quasi-experimental feasibility study
Source: Pilot Feasibility Stud. 2021 Nov 5;7:194. doi: 10.1186/s40814-021-00933-w (PMC8570014; doi:10.1186/s40814-021-00933-w)
Supplement: Supplementary file 2 — Additional file 2: TiDieR checklist. [file 40814_2021_933_MOESM2_ESM.docx]

**
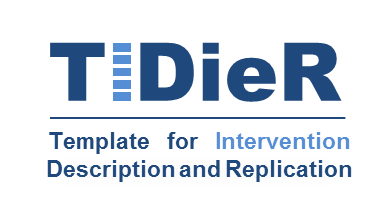
**

**Appendix 1: The TIDieR (Template for Intervention Description and Replication) checklist**

**Item 1: brief name**

Evaluation of the effect of Virtual Childbirth Preparation Courses on the Fear of Childbirth and Pregnancy Experience in Pregnant Women

**Item 2: why? (Rationale, theory or goal of the elements essential to the intervention)**

Childbirth preparation courses are an important component of the prenatal education and pregnant women are increasingly inclined to seek information from online sources.

The benefits of education during pregnancy include the correction of pregnancy and delivery misconceptions, improvement of mother's self-confidence regarding labor and childbirth, empowerment of women to select a safe delivery method, and a decrease in the need for analgesics during labor and childbirth. On the other hand, inadequate care during pregnancy leads to the preference of the cesarean section, postpartum depression, and challenges in accepting the maternal role.

One of the important educational principles in healthcare is the selection of an appropriate education method to have the active participation of individuals. Educational media plays an important role in teaching and forming positive behaviors at the community level. Some of the advantages of social media include the provision of services, such as health-related information at any time and location, online consultation, health-related question and answer, interactions between users, emotional and social support, and easy access regardless of age, level of education, race, and place of residence. Furthermore, social media are effective in shaping the attitudes of individuals through creating an interactive environment among group members.

## Item 3-4: what? (Materials used in the intervention, including those provided to participants or used in intervention delivery or in training of intervention providers)

**Materials**: The demographic characteristics questionnaire will be developed in two sections. The first part seeks personal information, such as age, level of education, as well as the occupational and economic status of couples. The second part asks about the history of pregnancy (i.e., the date of the last menstruation period, the expected date of delivery, and the recent pregnancy status).

The brief version of PES is available at:

<https://www.tandfonline.com/doi/abs/10.1080/01674820802546220>

The brief version of PES was first developed by DiPietro (2008) with 20 items. The first and second 10 items assess the uplifts and hassles among pregnant women. The items are scored based on a 4-point Likert scale ranging from not at all (0) to somewhat (1), quite a bit (2), and a great deal (3). The total scores for each section are within the range of 0-30, and the higher scores indicate uplifts and hassles. The reliability of the tool was measured using internal consistency and is estimated at 0.82 and 0.83 Cronbach’s alpha for uplifts and hassles, respectively. The reliability was also measured using the test-retest method for consistency across time and the results were 0.56-0.83. In Iran, the Persian translation of this tool was carried out by Hajifoghaha while its psychometric properties were determined by Ebadi. The face, content, and construct validity of PES have also been confirmed. In order to determine the reliability of the test, an internal consistency with a Cronbach alpha of 0.777 and 0.672 was used for uplifts and hassles, respectively. Moreover, in this regard, intraclass correlation coefficients of 0.712 and 0.672 were used for uplifts and hassles, respectively. This tool is valid in weeks 15-38 of gestation.

The WDEQ-A is available at:

<https://www.tandfonline.com/doi/abs/10.3109/01674829809048501>

The WDEQ-A was developed by Wijma (1998) and consists of 33 items which are scored based on a 6-point Likert scale ranging from ' not at all…' to 'extremely…'. The total scores were obtained within the range of 0-165 so that the scores of ≤ 37, 38 - 65, 66 - 84, and ≥ 85 were recognized as mild, moderate, severe, and clinical fear, respectively. Notably, reverse scoring was applied to items 2, 3, 6, 7, 8, 11, 12, 15, 19, 20, 24, 25, 27, and 31. The reliability of the results during the last trimester has been estimated at 0.89 Cronbach’s alpha, and the split-half reliability of the test has been obtained at 0.91. The Persian version of this questionnaire was developed by Abedi et al. in Iran, and the reliability of this scale has been reported at 0.64 Cronbach’s alpha for the whole pregnancy period.

**Procedures**: The first outcome of the present study is the improvement of pregnancy experience measured by the brief version of the pregnancy experience scale (PES) while version A of Wijma Delivery expectancy/experience questionnaire (WDEQ-A) measures the decreased FOC as the second outcome. In addition, the third and fourth outcomes are the birth preference and type of delivery, respectively. Data collection is shown in the flow diagram (Figure 1). Data will be collected at 3 time points via self-report and Telegram messages: Recruitment (demographic characteristics questionnaire, PES, WDEQ-A) ≈ 18-20 weeks (T1); 36-38 weeks (PES, WDEQ-A, and birth preference), (T2); the first few postpartum days (type of delivery), (T3).

**Item 5: who provided each aspect of the intervention?**

Training will be delivered by the research team, led by Leila Amiri-Farahani, Ph.D. of Reproductive Health, Associate Professor, Department of Reproductive Health and Midwifery, Faculty of Nursing and Midwifery, Iran University of Medical Sciences, Tehran, Iran.

Virtual education content will be designed by Seyedeh Robab Mousavi (MSc Student in Midwifery, Department of Reproductive Health and Midwifery, Faculty of Nursing and Midwifery, Iran University of Medical Sciences, Tehran, Iran) and Soghra omrani saravi (Distance education planning (Ph.D.), instructional Design consultant at E_learning Center of Iran University of Medical Sciences-Tehran-Iran). In addition, Seyedeh Robab Mousavi is responsible for training virtual childbirth preparation courses and in-person childbirth preparation courses will be provided by Milad Hospital midwives (It should be noted that the study researcher and all instructors of childbirth preparation courses have participated in a 60-hour instruction course held by the Ministry of Health and have obtained childbirth educator certification.).

**Item 6: how? The modes of delivery (face to face or by some other mechanism, such as internet or telephone) of the intervention and whether it was provided individually or in a group**

The participants in study groups A and B will be subjected to social media-based and in-person preparation training, respectively. Also, the control group will receives no education regarding childbirth preparation during pregnancy.

For the purposes of the study, a Telegram channel entitled “Virtual childbirth preparation courses” will be created for uploading the educational content. In addition, another Telegram group will be created with the same name in which the participants could ask their questions and receive answers.

On the other hand, subjects in the study group B will participate in eight 2-hour sessions during the 20-37 weeks of gestation. The educational sessions will be held at the hospital based on the guidelines of the Ministry of Health.

**Item 7: where? Type(s) of location(s) where the intervention occurred, including any necessary infrastructure or relevant features**

The virtually educational content will be provided based on the standard regulations of Iran and according to Mayer's principles of multimedia learning using text, picture, videocast, podcast, and MPEG4 (MP4) videos. Moreover, the maximum size of the educational files will be 50 MB in the form of 5-15-minute videos. In total, the educational contents will include six PDF files, 21 videos, four podcasts about relaxation and breathing techniques during labor, 14 videocasts, and 64 MP4 video files about exercise during and after pregnancy, massage, posisions related to labor, birth ball exercises and exercises after childbirth.

The educational sessions for study group B will be held at the Milad Hospital, Tehran, Iran based on the guidelines of the Ministry of Health. The virtually educational content will be provided based on the standard regulations of Iran and according to Mayer's principles of multimedia learning [17] using text, picture, videocast, podcast, and MPEG4 (MP4) videos. Moreover, the maximum size of the educational files will be 50 MB in the form of 5-15-minute videos.

**Item 8: when and how much? The number of times the intervention was delivered and over what period of time, including the number of sessions, their schedule and their duration, intensity or dose**

In 18-20 weeks of gestation, participants will be recruited and screened. The subjects in the study group B will participate in eight 2-hour sessions during the 20-37 weeks of gestation. In the study group A, to increase the quality of training and prevent the entire content from being sent at once, the content of each session is presented in sections that are loaded at the same time with study group B.

In addition, all participants will be followed up until delivery.

## Item 9: tailoring – if the intervention was planned to be personalised, titrated or adapted, then describe what, why, when, and how

This quasi-experimental study with a control group will be conducted on 165 primiparous women. The researcher will attend the prenatal clinic to perform sampling on a daily basis. The participants will be equally and not randomly allocated into two studies and one control group. The participants in study groups A and B will be subjected to social media-based and in-person preparation training, respectively. Also, the control group will receives no education regarding childbirth preparation during pregnancy.

The virtually educational content will be provided based on the standard regulations of Iran and according to Mayer's principles of multimedia learning using text, picture, videocast, podcast, and MPEG4 (MP4) videos. Moreover, the maximum size of the educational files will be 50 MB in the form of 5-15-minute videos.

In total, the educational contents will include six PDF files, 21 videos, four podcasts about relaxation and breathing techniques during labor, 14 videocasts, and 64 MP4 video files about exercise during and after pregnancy, massage, posisions related to labor, birth ball exercises and exercises after childbirth.

The questions asked on the Telegram group will be replied by the researcher at least twice a day. During the last weeks of pregnancy and after uploading all files, the educational content regarding labor and childbirth preparation methods will be reviewed.

In the study group B, in each session, one hour will be allocated to teaching theoretical content followed by 45 min of stretching, breathing, and relaxing techniques, as well as practical strategies for posture correction, and massage therapy education. The participants are further will provide with 15 min to raise any questions which will be replied by the researcher.

To increase the generalizability of the results, another group will be considered as the control group, the members of which will receive no educational courses about pregnancy and childbirth preparation whether through Telegram or in person. It is important to note that all three groups will receive perinatal care.

## Item 10: modifications – if the intervention was modified during the course of the study, describe the changes (what, why, when and how)

The present study is a Protocol for a Quasi-Experimental Clinical Trial and does not have any modifications due to non-implementation.

## Item 11: how well (planned) – describe how, by whom and if any strategies were used to maintain or improve fidelity

To ensure the accurate learning of breathing and relaxation techniques, two 2-hour sessions will be held for the in-person training of the study group A at the hospital along with the fifth and eighth sessions of the study group B. In addition, in order to ensure that the messages are read by the participants, before joining the group, they will be asked to share their last seen status with the researcher. Moreover, they will be required to become online at least once a day to watch the videos or read the messages. The last seen time of the participants will be controlled by the researcher, and those who are not online, do not see messages, or do not give feedback for seven days are initially contacted through message. In case of receiving no answer, the researcher will contact them through a phone call to ask for the reason. If needed, another cell phone number will be obtained to re-send the Telegram messages to that number. Otherwise, the subject will be excluded from the study.

## Item 12: how well (actual) – the extent to which the intervention was delivered as planned

The present study is a protocol for a quasi-experimental clinical trial and has not yet been implemented.
